# Supplementary material for: A continuous-time state-space model for rapid quality control of argos locations from animal-borne tags
Source: Mov Ecol. 2020 Jul 17;8:31. doi: 10.1186/s40462-020-00217-7 (PMC7368688; doi:10.1186/s40462-020-00217-7)
Supplement: Supplementary file 1 — Additional file 1 Supplementary figures. [file 40462_2020_217_MOESM1_ESM.pdf]

Additional Files

Additional file 1 — Supplementary figures.

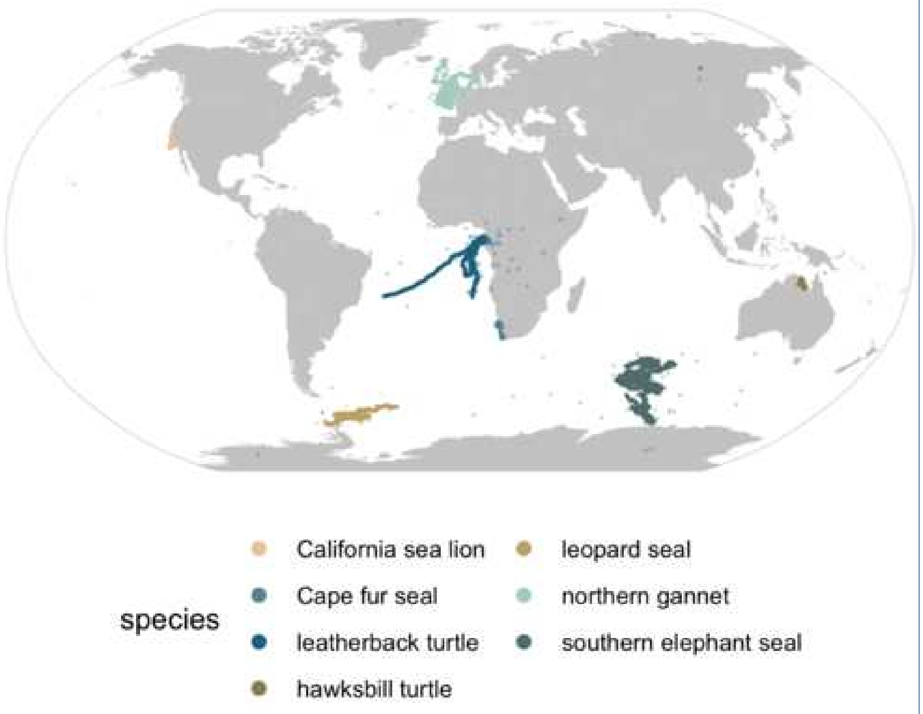

Figure S1 Argos tracking data by species. Locations displayed are unfiltered Argos Least-Squares data.

The majority of visible ellipses are highly compressed on their semi-minor axes for both Argos data types. The dashed blue track line helps highlight the outlier location (upper right) that clearly has a vastly under-estimated error ellipse. Application of the Kalman smoother (b) did not reduce this outlier or improve its uncertainty estimate.

The majority of ellipses are highly compressed on their semi-minor axes for both Argos data types. The dashed blue track line helps highlight the outlier location (lower right) that has an error ellipse with under-estimated semi-minor axis. Application of the Kalman smoother (b) did not reduce this outlier or improve its uncertainty estimate.

The majority of ellipses are highly compressed on their semi-minor axes for both Argos data types. Application of the Kalman smoother (b) generally reduced the size of error ellipses but did not alter the highly compressed nature of the ellipse semi-minor axes.

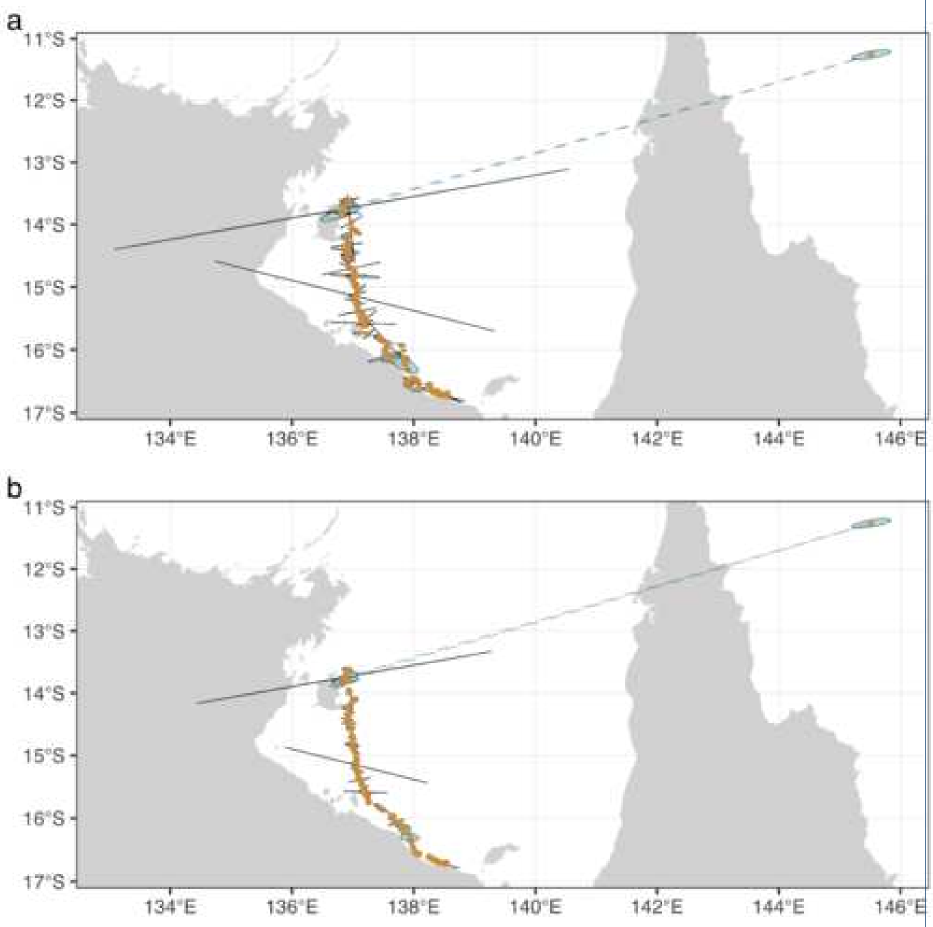

Figure S2 Argos error ellipses for a representative hawksbill turtle track. The maps display (a) Argos Kalman filter- and (b) Argos Kalman smoother-measured locations (beige) and error ellipses (light blue with black borders). Temporally sequential but spatially distant locations are connected by dashed blue lines.

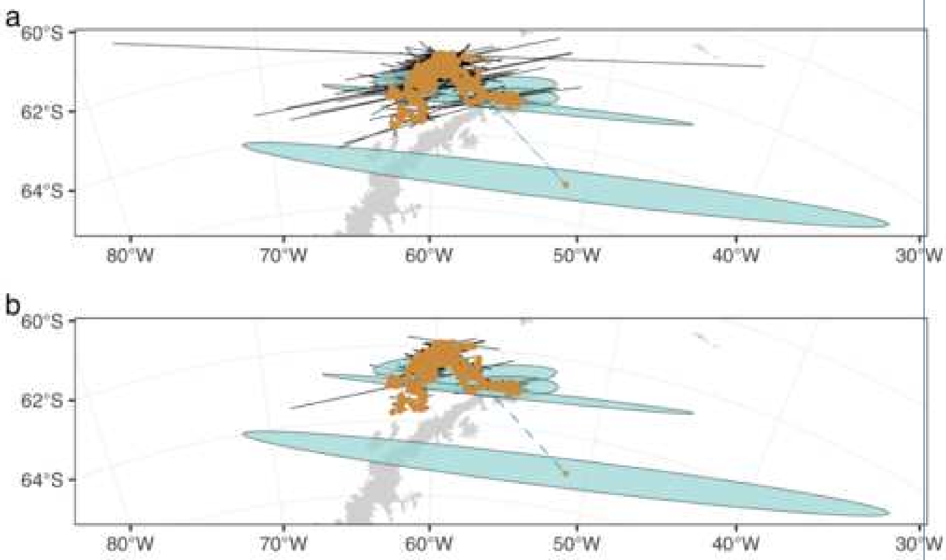

Figure S3 Argos error ellipses for a representative leopard seal track. The maps display (a) Argos Kalman filter- and (b) Argos Kalman smoother-measured locations (beige) and error ellipses (light blue with black borders). Temporally sequential but spatially distant locations are connected by dashed blue lines.

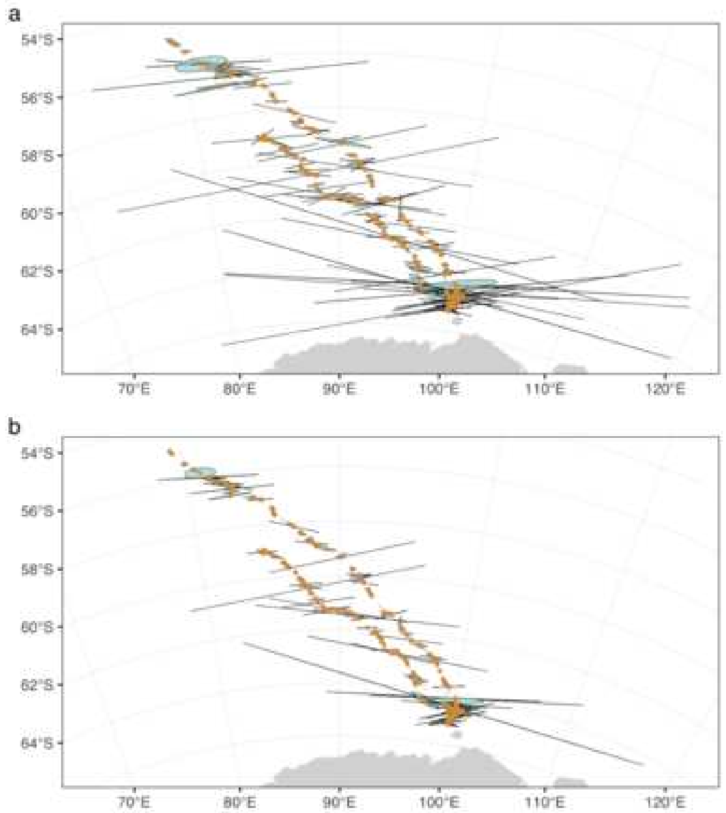

Figure S4 Argos error ellipses for a representative southern elephant seal track. The maps display (a) Argos Kalman filter- and (b) Argos Kalman smoother-measured locations (beige) and error ellipses (light blue with black borders).

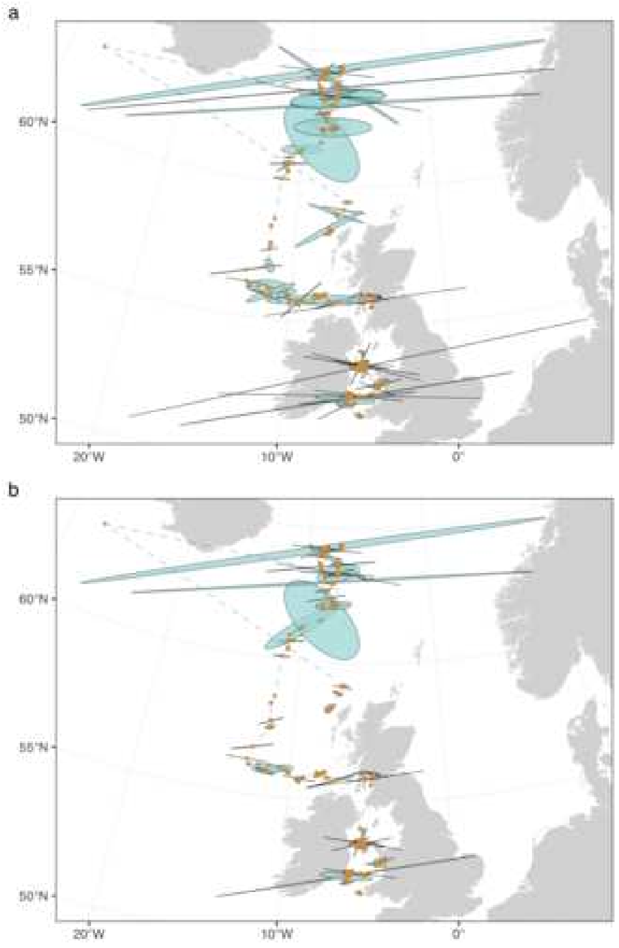

Figure S5 Argos error ellipses for a representative northern gannet track. The maps display (a) Argos Kalman filter- and (b) Argos Kalman smoother-measured locations (beige) and error ellipses (light blue with black borders). Temporally sequential but spatially distant locations are connected by dashed blue lines.

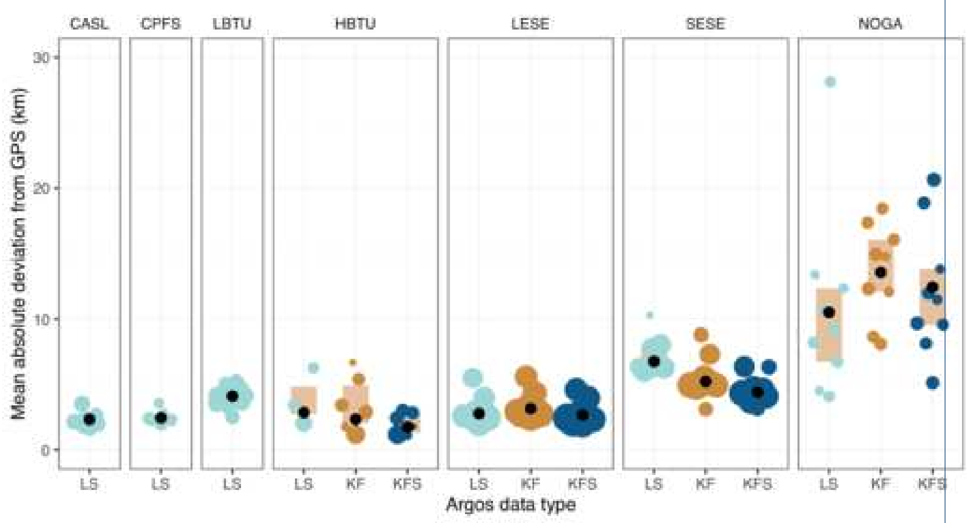

Figure S6 Root mean squared distance (RMSD in km) between state-space model-estimated locations and their corresponding temporally closest GPS locations within ± 10 min, by Argos data type and species. Size of RMSD points is proportional to $\sqrt{n}$ distances between temporally matched pairs of model-estimated and GPS locations. Black points are n-weighted means by species and data type.
